# Patterns of Federal Lobbying by the Hospital Industry

**DOI:** 10.1001/jamahealthforum.2026.0117

**Published:** 2026-03-13

**Authors:** Olivia Korostoff-Larsson, Caroline Shore, Lauren A. Taylor

**Affiliations:** 1 Department of Population Health, NYU Grossman School of Medicine, New York, New York; 2 NYU Wagner School of Public Policy, New York University, New York, New York

## Abstract

**Importance:**

Hospitals are major stakeholders in US health policy yet receive less scholarly attention than other health care industries, such as pharmaceuticals and health professionals. Understanding their federal lobbying activity is critical to evaluating how hospitals shape health policy.

**Objective:**

To characterize federal lobbying by the hospital industry in 2024, including health systems and hospital associations, and to examine the concentration of spending and use of internal vs external lobbyists.

**Design, Setting, and Participants:**

This descriptive cross-sectional study used 2024 federal lobbying disclosure data filed under the Lobbying Disclosure Act of 1995, compiled by OpenSecrets, a nonprofit website that standardizes and publishes data on political spending. and included all organizations categorized as hospitals or hospital associations that reported federal lobbying activity in 2024, along with their affiliated lobbying firms.

**Exposure:**

Organization type (nonprofit, for-profit, and private equity–owned).

**Main Outcomes and Measures:**

The primary outcomes were total and mean lobbying expenditures, and use of internal vs external lobbyists. Lobbying firm outcomes included total hospital industry earnings and clients and the share of total earnings and clients attributable to the hospital industry.

**Results:**

In 2024, 355 hospital-related organizations reported $116.13 million in federal lobbying expenditures. Hospitals and health systems accounted for $70.56 million (60.7%), with the remainder from hospital associations. Eighteen organizations spent more than $1 million, including 12 health systems and 6 hospital associations. For-profit and private equity–owned systems comprised 8.5% of all lobbying health systems but 27.8% of the highest spenders (5 of 18 organizations). However, overall for-profit and nonprofit spending mirrored their share of US community hospitals. Nearly all hospitals and health systems (283 of 295 organizations [94.9%]) employed external lobbying firms. Lobbying by for-profit systems was highly concentrated among a few large organizations, while nonprofit lobbying was diffuse.

**Conclusions and Relevance:**

This cross-sectional study found that hospitals and affiliated organizations are major participants in federal health policy lobbying, relying on a small number of professional firms, with large health systems often outspending their state hospital associations. These patterns suggest lobbying influence is concentrated among well-resourced organizations and that not all hospitals have an equal voice in federal policymaking.

## Key Points

**Question:**

Which hospitals and hospital associations are most active in federal lobbying, who lobbies on their behalf, and what does this reveal about hospitals’ role in shaping health policy?

**Findings:**

In this cross-sectional study of federal lobbying disclosures from 355 hospital-related organizations, the hospital industry spent $116.13 million on federal lobbying in 2024, with 60.7% from hospitals and the remainder from associations. Of all hospitals, 94.9% employed professional lobbying firms, many outspent their state hospital associations, and for-profits were overrepresented among top spenders.

**Meaning:**

This cross-sectional study found that hospitals invest substantially in lobbying, suggesting they are not merely influenced by health policy but actively shape it.

## Teaser

This cross-sectional study quantifies federal lobbying expenditures by the hospital industry in 2024 and compares variations in lobbying expenditures by hospital ownership status.

## Introduction

Hospitals, and particularly hospital associations, have a long track record of exerting substantial influence on US health policy. In 2010, the American Hospital Association (AHA) was among the most powerful interests supporting passage of the Patient Protection and Affordable Care Act, against considerable political objections from Republican members of Congress.

More recently, the One Big Beautiful Bill prompted intensive lobbying from the hospital industry in opposition to proposed restrictions on Medicaid provider taxes, provider assessments used to finance states’ Medicaid share and leverage federal matching funds. The AHA reported record third-quarter 2025 lobbying expenditures and mobilized hundreds of hospital leaders in Washington, DC.^1,2^ Hospital lobbying was successful in the House of Representatives, where lawmakers declined to cut existing provider taxes and instead adopted a moratorium on new taxes. The Senate, however, advanced a more restrictive version requiring many states to lower existing provider tax rates despite sustained hospital lobbying, illustrating both the industry’s political influence and its limits in the current climate.^1^

These episodes highlight the role hospitals play in shaping government policy at the same time as their finances and operations are strongly influenced by policy. In 2024, the hospital and nursing home industry spent more than $131 million on federal lobbying, making it the second highest spender in the health care sector after the pharmaceutical and health products industry. These funds are used to influence a wide range of policy issues affecting hospital operations and finances, including site-neutral payment reform, surprise billing, and the 340B Drug Pricing Program. Moreover, hospital assets have grown substantially over time, with a widening gap between wealthy and poor hospitals.^3^ This may translate into unequal ability to engage in and influence policymaking.

Despite the hospital industry’s substantial and potentially uneven capacity for political engagement, it has received limited attention in academic research. We use the term *hospital industry* to refer to hospitals, health systems, and hospital associations, one part of the broader health care sector. Most prior research on health care sector lobbying has focused on pharmaceutical and health product manufacturers^4,5,6^ or on health professional organizations.^7^ A 2022 study by Schpero et al^8^ showed that lobbying by health care providers, including hospitals, health care professionals, nursing homes, and trade associations, grew substantially between 2000 and 2020, nearly matching the pace of increases in pharmaceutical and health product lobbying. However, hospitals were not analyzed separately, leaving open questions about how hospital-specific lobbying contributes to health policy formation. In addition, little is known about how hospitals organize their lobbying activity, including whether they rely on internal government-relations staff or contract with external lobbying firms. Reliance on external firms raises potential principal-agent concerns and motivates examination of consolidation and concentration of influence among a small set of firms.^9^

To better understand this underexamined but influential industry, we conducted a descriptive cross-sectional analysis of 2024 hospital lobbying activity using data from OpenSecrets, an independent nonprofit that collects, standardizes, and publishes information on money in US politics to support transparency and public accountability. We analyzed which organizations spent the most, which lobbying firms were most active, and how lobbying activity varied by hospital ownership status. This analysis aims to characterize hospital industry influence on US federal health policy.

## Methods

We conducted a descriptive cross-sectional study of 2024 federal lobbying expenditures by hospitals and hospital associations using data from OpenSecrets, which compiles quarterly filings submitted by lobbying firms or organizations with in-house lobbyists under the Lobbying Disclosure Act of 1995 to the US Senate Office of Public Records. These filings contain good faith estimates of lobbying expenditures and identify client organizations, affiliated lobbying firms, and individual lobbyists. This study followed the Strengthening the Reporting of Observational Studies in Epidemiology (STROBE) reporting guideline. In accordance with 45 CFR § 46, no institutional review board approval was sought and no informed consent was required because the study is not human participants research.

OpenSecrets organizes lobbying expenditures into 10 major sectors, each divided into specific industries. This analysis focused on the hospitals and nursing homes industry within the broader health sector. On July 23, 2025, we downloaded a CSV file from the OpenSecrets industry profile summary page^10^ listing all clients in the hospitals and nursing homes industry that reported federal lobbying activity in 2024, along with their total expenditures per client. It also lists whether a client lobbied for any of its subsidiaries or affiliates.

We then categorized organizations into 5 groups: nonprofit health systems, for-profit health systems, private equity (PE)–owned health systems, state hospital associations, and national hospital associations. Public hospitals were grouped together with nonprofits. We examined lobbying patterns by hospital ownership type because current policy debates may differentially affect nonprofit, for-profit, and PE-owned hospitals. For example, recent legislation introduced by Senator Elizabeth Warren specifically proposes heightened oversight and restrictions on PE-owned health care entities.^11^ Classification was based on organizational websites, OpenSecrets profiles, and web searches. Entities not primarily representing hospitals (eg, nursing homes or mental health and addiction treatment centers) were excluded to focus the analysis on hospital-related lobbying. A complete list of organizations, along with detailed definitions of each category, is provided in eTable 1 in Supplement 1.

### Statistical Analysis

Primary outcomes were total and mean lobbying expenditures and use of internal and external lobbyists by hospital ownership status (nonprofit, for-profit, or PE-owned). We first extracted total 2024 lobbying expenditures for each included organization from the downloaded dataset. We ranked organizations by spending and calculated total and mean annual spending for each organizational category. For the top 20 spenders, we looked at lobbying report images published on OpenSecrets to extract which agencies they lobbied. To determine whether organizations relied on internal or external lobbyists, we manually extracted records on each organization’s OpenSecrets client profile pages for lobbyists. This page lists each lobbyist employed by the organization, their lobbying firm, and the total amount of the associated lobbying contract. We compiled all of the lobbyists used and classified each as internal if their firm was the same as the client organization, indicating direct employment, or external, if affiliated with a separate lobbying firm contracted to represent the client.

Lobbying firm outcomes included total hospital industry earnings and clients and the share of total earnings and clients attributable to the hospital industry. For each lobbying firm that was hired by at least 1 hospital industry client in 2024, we collected information on earnings and number of clients from their OpenSecrets lobbying firm profile pages. These totals are explicitly listed by OpenSecrets and served as the denominators for proportion calculations. We then downloaded the CSV of all clients retaining each firm, identified hospital-industry clients, and summed their associated payments. Hospital-industry earnings and clients were divided by the firm’s total earnings and client count to calculate the proportion attributable to the hospital industry. Firms were then ranked by hospital-industry earnings, and summary statistics were compiled for the top 20 firms.

To examine state-level variation in hospital association lobbying, we attributed each association’s reported federal lobbying expenditures to the states it represents. There was only 1 multistate hospital association, representing hospitals in New York, New Jersey, Rhode Island, and Connecticut; its full reported spending amount was attributed to each of those 4 states to reflect the total lobbying resources available to hospitals in each. State-level lobbying intensity was defined as hospital association lobbying expenditures per hospital, calculated as total attributed spending divided by the number of hospitals in the state, using data from the American Hospital Directory.^12^ States were ranked by per-hospital spending. Analyses were conducted using Microsoft Excel Version 16.89.1 (Microsoft Corp).

## Results

In 2024, 355 hospitals and hospital associations devoted more than $116.13 million to federal lobbying (Figure and Table 1). Of this total, 295 hospitals and health systems, representing more than 3200 individual hospitals, accounted for $70.56 million (60.7%), with spending per organization ranging from $5000 to $5.50 million (mean [SD], $239 177 [$466 855]; median [IQR], $120 000 [$80 000-$225 245]). Sixty hospital associations at national and state levels spent $45.58 million (39.2%) (eTable 2 and eTable 3 in Supplement 1). All organizations reported lobbying Congress, with additional efforts directed toward federal agencies. Among the top 20 spenders in 2024, the most frequently targeted agencies were the Department of Health and Human Services (18 organizations [90.0%]), the Centers for Medicare & Medicaid Services (17 organizations [85.0%]), the White House (9 organizations [45.0%]), and the Health Resources and Services Administration (9 organizations [45.0%]).

**Figure.**
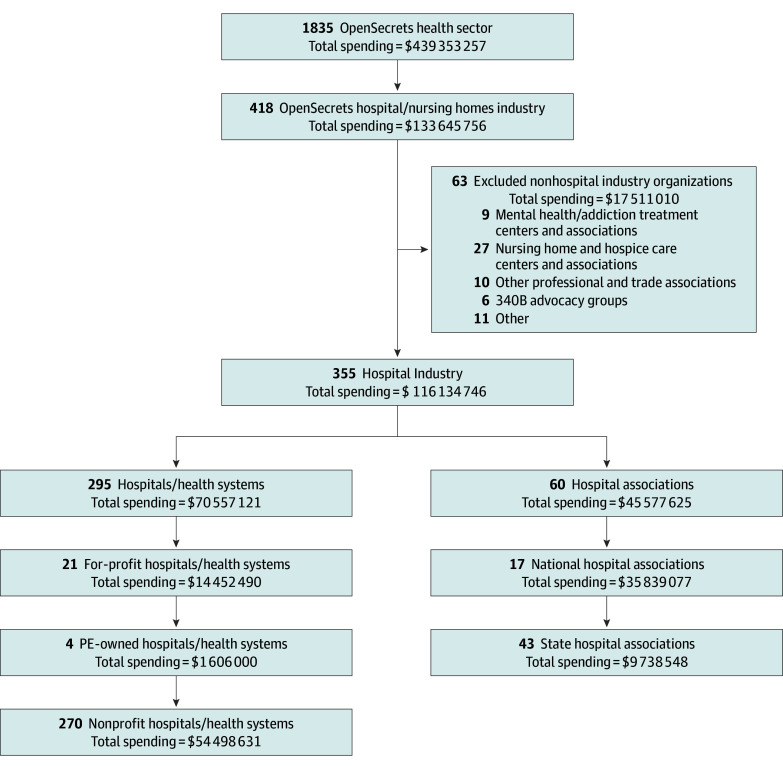
Flow Diagram of Hospital Industry Dataset Derived From OpenSecrets Health Sector PE indicates private equity.

**Table 1.** Annual Mean and Total Federal Lobbying Expenditures by Health Care Industry, 2020-2024

Industry	Expenditures by year, $					Percent change
	2020	2021	2022	2023	2024	
Total lobbying spending						
Pharmaceuticals and health products	318 993 801	364 969 269	380 445 634	385 353 389	387 529 822	21
Hospitals and nursing homes^a^	117 679 880	121 292 282	125 574 977	134 188 475	133 292 078	13
Health services and HMOs	109 758 087	123 721 039	128 240 718	129 657 968	118 027 295	8
Health professionals	88 886 950	90 371 333	98 144 942	96 911 743	99 918 474	12
Miscellaneous health	7 110 537	7 797 158	8 968 100	8 985 439	12 132 657	71
Health sector total	642 429 255	708 151 081	741 374 371	755 097 014	750 838 554	17
Mean (SD) lobbying spending (per client spending >$0)						
Pharmaceuticals and health products	588 549 (1 716 945)	620 696 (1 850 428)	654 812 (1 822 455)	664 402 (1 944 468)	729 811 (2 028 386)	24
Health professionals	388 153 (1 278 506)	398 112 (1 306 113)	426 717 (1 397 846)	423 195 (1 401 703)	454 175 (1 645 871)	17
Hospitals and nursing homes	306 458 (1 231 718)	311 805 (1 240 972)	319 529 (1 313 857)	344 958 (1 520 481)	337 292 (1 447 990)	10
Health services and HMOs	285 086 (689 088)	303 983 (765 966)	314 315 (781 702)	328 248 (920 968)	337 221 (783 935)	18
Miscellaneous health	107 735 (136 580)	119 956 (147 227)	126 311 (163 742)	112 318 (168 563)	102 819 (152 474)	−5
Health sector total	400 018 (609 687)	422 525 (639 860)	440 508 (639 179)	451 343 (678 042)	465 204 (744 355)	16

Abbreviation: HMO, health maintenance organization.

^a^ OpenSecrets combines nursing homes and hospitals into a single category. We disaggregated these data and found that hospitals make up the majority of the category’s total and average spending. In 2024, hospitals and hospital associations made up 87.1% ($116 134 746 of $133 292 078) of the total hospitals and nursing home industry spending.

### Hospital Ownership Status

Lobbying patterns varied by health system ownership status (Table 2). Among the 295 hospitals and health systems that filed lobbying disclosures in 2024, nonprofit systems accounted for 91.5% of the lobbying organizations (270 of 295 organizations) and 77.2% of total 2024 federal lobbying expenditures ($54.50 million of $70.56 million). Nonprofit organizations had the lowest mean (SD) spending ($201 847 [$286 922]). Together, for-profit and PE-owned systems comprised 8.5% of all lobbying health systems; for-profit systems made up 7.1% of lobbying organizations (21 of 295 organizations) but 20.4% ($14.45 million) of total spending, with the highest mean (SD) expenditure ($688 214 [$1 349 271]) and greatest use of internal lobbyists (6 of 21 organizations [28.5%]), while PE-owned systems accounted for 1.3% of lobbying organizations (4 of 295 organizations) and 2.3% ($1.61 million) of spending, relying exclusively on external lobbying firms.

**Table 2.** Lobbying Expenditures, Hospital Share, and Use of Internal vs External Lobbyists by Health System Ownership Status

Health system ownership status	No. %			Spending, $		Organizations, No./total No. (%)^b^	
	Lobbying organization (n = 295)	Total lobbying spending, $(n = 70 557 121)	Community hospitals in US (n = 5112)^a^				
				Mean (SD)	Median (IQR)	With internal lobbyists	With external lobbyists
Nonprofit^c^	270 (91.5)	54 498 631 (77.2)	3898 (76.3)	201 847 (286 922)	120 000 (80 000-209 050)	45/270 (16.7)	259/270 (95.9)
For-profit	21 (7.1)	14 452 490 (20.4)	1214 (23.7)^d^	688 214 (1 349 271)	120 000 (80 000-310 000)	6/21 (28.5)	20/21 (95.2)
PE-owned	4 (1.3)	1 606 000 (2.3)		401 500 (518 468)	215 000 (87 500-529 000)	0	4/4 (100.0)
Total	NA	NA	NA	239 177 (466 855)	120 000 (80 000-225 245)	51/295 (17.3)	283/295 (95.9)

Abbreviations: NA, not applicable; PE, private equity.

^a^ Numbers of community hospitals in the US published by the American Hospital Association, 2025.^13^

^b^ These columns do not total 100% because some organizations have both internal and external lobbyists.

^c^ Nonprofit includes government owned and operated.

^d^ This number is all for-profit community hospitals. The American Hospital Association data do not distinguish between for-profit and PE-owned hospitals.

The share of total federal lobbying spending attributable to nonprofit and for-profit (including PE-owned) hospitals closely reflects their representation among US community hospitals overall (Table 2).^13^ Investor-owned hospitals constituted 23.7% of community hospitals in the US (1214 of 5112 hospitals) and 22.7% of federal lobbying spending ($16.06 million). Nonprofit and government hospitals accounted for 76.3% of community hospitals (3898 of 5112 hospitals) and 77.2% of expenditures ($54.50 million).

### Top Spenders

There were 18 organizations that spent more than $1 million on federal lobbying in 2024, including 12 health systems and 6 hospital associations (4 national and 2 state) (Table 3). For-profit systems comprised only 7.1% of the dataset yet represented 22.2% of the top-spending organizations (4 of 18 organizations). Also among the top spenders was the Federation of American Hospitals, a national association representing the interests of for-profit hospitals, and one PE firm, Apollo Global Management, which lobbied through its subsidiary health systems, Lifepoint Health and Kindred Healthcare. Together, for-profit and PE-owned systems comprised 27.8% of the highest spenders.

**Table 3.** Hospital Industry Organizations With More Than $1 million in Reported Federal Lobbying Expenditures in 2024

Client or parent organization	Subsidiary and affiliates	Hospitals represented, No.^a^	Total 2024 spending, $	Type of organization
American Hospital Association	NA	5000	24 110 000	National hospital association
Select Medical Holdings	NA	100	5 500 000	For-profit health system
Children’s Hospital Association	NA	220	4 170 000	National hospital association
HCA Inc	NA	180	3 010 000	For-profit health system
Advocate Health	Advocate Health, Atrium Health, and Atrium Wake Forest Baptist	69	2 420 000	Nonprofit health system
Federation of American Hospitals	NA	970	2 380 000	National hospital association
Tenet Healthcare	NA	50	2 200 000	For-profit health system
Ascension Health	NA	94	2 163 000	Nonprofit health system
America’s Essential Hospitals	NA	300	1 960 000	National hospital association
Greater New York Hospital Association	NA	230	1 890 000	State hospital association
California Hospital Association	NA	400	1 530 000	State hospital association
Trinity Health	BayCare Health System and Trinity Health	90	1 646 232	Nonprofit health system
Ochsner Health System	Ochsner Health System and Ochsner Clinic Foundation	46	1 630 000	Nonprofit health system
Mass General Brigham	NA	16	1 223 000	Nonprofit health system
Encompass Health	NA	150	1 195 000	For-profit health system
Apollo Global Management	Lifepoint Health and Kindred Healthcare	150	1 156 000	PE-owned health system
Mayo Clinic	NA	16	1 050 000	Nonprofit health system
UPMC Health System	NA	40	1 030 000	Nonprofit health system

Abbreviations: NA, not applicable; PE, private equity.

^a^ Reflects owned hospitals for systems and member hospitals and facilities for associations. Counts are approximate.

The AHA, which represents 5000 hospitals and health systems in the US, dominates hospital-industry lobbying. The AHA spent $24.11 million on lobbying in 2024, more than 4 times the next highest spender. Among state hospital associations, only 2 associations spent more than $1 million on lobbying in 2024.

Many health systems and hospitals spent more on federal lobbying individually than the state hospital associations representing their home states. For example, Mass General Brigham, with 16 hospitals, spent $1.22 million on federal lobbying in 2024, compared with only $80 000 by the Massachusetts Health and Hospital Association, which represents 70 hospitals. Ascension Health, with 94 hospitals across 10 states, reported $2.16 million in lobbying, which is more than the aggregate spending of the hospital associations in those states ($1.52 million). The same is true for Mayo Clinic, which spent $1.05 million, more than the aggregate spending of the hospital associations in Arizona, Florida, and Minnesota ($331 481).

### Hospital Association Lobbying

Hospital associations at the national and state levels play a substantial role in hospital lobbying. In 2024, they collectively reported $45.58 million in federal lobbying expenditures, accounting for 39.2% of total spending in the hospital industry. These organizations represented a wide range of hospital interests, including for-profit, safety-net, pediatric, rural, and specialty hospitals (eTable 2 in Supplement 1).

State and regional hospital associations from across the country varied widely in federal lobbying activity. A regional association representing hospitals in New York, New Jersey, Rhode Island, and Connecticut reported the highest spending at $1.89 million, followed by the state hospital association in California ($1.53 million) (eTable 3 in Supplement 1). Many states, particularly in the Mountain West and Deep South, reported minimal or no spending by a state or regional hospital association. State-level variation in hospital association lobbying expenditures is provided in eTable 4 and the eFigure in Supplement 1.

### Lobbying Firms

Use of external lobbyists was nearly universal among lobbying health systems and hospitals (283 of 295 organizations [95.9%]), reinforcing the central role of professional lobbying firms in representing hospital interests in Washington, DC. In 2024, 169 lobbying firms filed reports on behalf of hospital industry clients under the Lobbying Disclosure Act. Of these, 71 firms had more than 1 client, 23 had 5 or more clients, and just 7 firms had 10 or more clients in the industry. Table 4 lists the top 20 firms in terms of hospital industry earnings.

**Table 4.** Lobbying Firms With the Highest 2024 Earnings From Hospital Industry Clients, Including Share of Total Clients and Revenue in 2024

Lobbying firm	No./total No. (%)	
	Hospital-industry earnings, $	Hospital-industry clients
Merchant, McIntyre & Associates	2 040 000/6 650 000 (30.7)	24/87 (27.6)
Brownstein, Hyatt et al	2 016 000/67 780 000 (3.0)	10/310 (3.2)
Cornerstone Government Affairs	1 990 000/48 210 000 (4.1)	14/333 (4.2)
Strategic Health Care	1 934 000/2 212 000 (87.4)	21/57 (36.8)
Alston & Bird	1 820 000/10 400 000 (17.5)	15/70 (21.4)
BGR Group	1 750 000/45 080 000 (3.9)	9/224 (4.0)
Mehlman Consulting	1 720 000/26 240 000 (6.6)	8/134 (6.0)
Keller Partners & Co	1 700 000/3 960 000 (42.9)	17/47 (36.2)
Carmen Group	1 452 000/4 453 000 (32.6)	4/23 (17.4)
DeBrunner & Associates	1 355 095/1 550 732 (87.4)	10/14 (71.4)
Thorn Run Partners	1 280 000/30 050 000 (4.3)	7/228 (3.1)
McDermottPlus	1 110 000/4 390 000 (25.3)	7/33 (21.2)
Manatt, Phelps & Phillips	1 042 500/2 392 500 (43.6)	5/28 (17.9)
Holland & Knight	1 040 000/49 710 000 (2.1)	6/318 (1.9)
Cassidy & Associates	960 000/25 810 000 (3.7)	5/170 (2.9)
Welsh Rose LLC	950 000/2 000 000 (47.5)	4/9 (44.4)
Powers, Pyles et al	890 000/4 589 000 (19.4)	7/47 (14.9)
Nathanson & Hauck	880 000/3 120 000 (28.2)	3/12 (25.0)
Hall, Render et al	856 898/1 693 898 (50.6)	10/38 (26.3)
Daschle Group	770 000/4 810 000 (16.0)	4/40 (10.0)

These firms varied substantially in their degree of industry specialization. Among the top-earning firms, Brownstein Hyatt ($2.02 million of $67.78 million million [3.0%]), Cornerstone Government Affairs ($1.99 million of $48.21 million [4.1%]), and BGR Group ($1.75 million of $45.08 million [3.9%]) derived only a small fraction of their overall earnings from hospitals. In contrast, firms such as Strategic Health Care ($1.93 million of $2.21 million [87.4%]), DeBrunner & Associates ($1.36 million of $1.55 million [87.4%]), and Hall, Render ($0.86 million of $1.64 million; [50.6%]) were far more specialized, with much larger shares of their revenue and client base tied to hospitals.

## Discussion

The available data in this cross-sectional study reveal important insights into how hospitals engage with the policy process. Hospitals and health systems are frequently characterized as passive recipients of health policy. While it is true that hospitals’ behavior is constrained by government policies, this framing obscures the important role they play as active participants in the policymaking process. In short, hospitals are both policy takers and shapers. While this study does not specifically capture these different mechanisms, shaping policy may include agenda setting via advocacy agendas, making support or opposition to ideas known to policymakers, drafting boilerplate policy language, and providing feedback on proposed rules.

Within hospital lobbying, 3 findings merit highlighting. First, we observed distinct lobbying patterns by hospital ownership type. Disclosure of lobbying activity by nonprofit and for-profit hospitals roughly mirrored their overall share of community hospitals in the US, but the intensity and concentration of lobbying spending across organizations differed notably between these 2 ownership types. Nonprofit hospitals and health systems constituted 91% of lobbying entities yet generated 77% of total expenditures. Conversely, for-profits and PE-owned hospitals comprised just 8% of organizations but generated 23% of total expenditures. For-profits were also overrepresented among the biggest spenders in the hospital industry; five of the 18 organizations (27.7%) that spent more than $1 million in 2024 were for-profit or PE-owned (Select Medical, HCA Inc, Tenet, Encompass, and Apollo Global Management), despite constituting only 8% of the overall dataset. This finding indicates that for-profit lobbying is highly concentrated among a small number of large corporate systems, while nonprofit lobbying is much more diffuse. Higher per-organization lobbying expenditures among for-profit hospitals could reflect greater discretionary spending capacity coupled with higher price or quantity of lobbying activity purchased, compared with nonprofits. One explanation for this may be that for-profit-specific policy priorities receive less emphasis within broad hospital trade associations such as the AHA, prompting greater reliance on direct lobbying to secure an audience for for-profit health system concerns.

Second, some state associations, most notably California and New York, rank among the top federal spenders and comfortably outspend their member health systems. However, large health systems often spend more on federal lobbying than the state hospital associations of which they are a part; this raises important questions about the concentration of political voice among a relatively small number of wealthier hospitals and health systems, potentially overshadowing the associations intended to represent hospitals collectively. Consistent with this interpretation, Schpero et al^8^ found that the top 10% of provider organizations accounted for nearly 60% of total lobbying expenditures, again illustrating a pattern of influence concentrated among large, well-resourced institutions. At the same time, the observed disparity may reflect differing priorities; state associations may focus more on state-level policy, while these major systems are more active federally. Moreover, higher federal lobbying by individual health systems often occurs in states with more competitive health care markets, where several domains of policymaking action (eg, not only Medicaid, but Certificate of Need, antitrust, and union contracting) invite lobbying. In states where this is the case, increased federal lobbying could reflect spillover from extensive state-level advocacy.

Lastly, most hospitals and hospital associations rely on third-party lobbyists. The choice between external and internal lobbyists has been described as a trade-off between agency and access: outside firms often provide greater reach and connections, but may bring their own agenda.^14^ As Rogan Kersh observed, lobbyists are not neutral messengers but “political actors in their own right” who can decide which issues to emphasize and even advance interests of their own.^15^ These dynamics may be particularly salient when lobbying firms represent multiple hospital clients with differing interests, such as large health systems and community hospitals, creating the potential for lobbyists to prioritize the concerns of better-resourced clients in the hopes of future business. While lobbying can facilitate the transmission of important information from frontline health care to policymakers, this signal may be filtered through lobbyists’ judgments about which messages to advance and with what intensity. Notably, only 7 states require disclosure of conflicting interests among lobbyists, according to a 2022 analysis.^14^ Internal lobbyists may align more closely with hospital values, but often lack the political capital of multiclient firms, leaving hospitals to balance access against control of their agenda.

It is tempting to think of the $116 million in spending by hospitals on federal lobbying in terms of the opportunity costs it presents. While such resources could conceivably be redirected toward patient care or other ends, lobbying is an integral feature of the political landscape in which US hospitals function. In some cases, hospital lobbying likely serves the public good by informing policymakers of on-the-ground realities they have no other means of accessing. What our findings highlight is that this political voice is not distributed equally across hospital types. Lobbying expenditures are concentrated among large, well-resourced systems and are often channeled through a relatively small set of professional lobbying firms with established connections to policymakers. Small, stand-alone, community-based hospitals are a diminishing presence in the industry and are also overshadowed in how they are represented in lobbying activity. These imbalances shape which perspectives are most likely to be heard and responded to in federal policymaking. Future policy inquiry may consider mechanisms to promote more representative participation across different types of hospitals.

### Limitations

This study has limitations. Lobbying disclosures are based largely on self-reported filings, as required by federal regulation and aggregated through sources like OpenSecrets. These disclosures capture relationships with branches of federal government, spending, and broad-issue areas, but most often do not provide details regarding positions on a specific issue, bill, or regulation. As a result, while we could observe spending patterns, our ability to assess the actual impact of hospital lobbying on specific policy issues was limited. In addition, due to lobbying registration thresholds under the Lobbying Disclosure Act, spending less than $12 500 per quarter does not have to be reported, and so this study provides a systematic underestimate of lobbying expenditures.^16^ Further, this analysis reflects a single year (2024) of lobbying activity and is not able to contextualize the observed patterns within longitudinal trends.

## Conclusion

This cross-sectional study found that hospitals and their affiliated organizations are major participants in federal health policy lobbying, largely relying on a small number of professional lobbying firms. Health systems often outspent their state hospital associations, suggesting concentrated influence among large, well-resourced organizations. For-profit lobbying was especially concentrated among the biggest corporate actors. These patterns suggest that not all hospitals have an equal voice in federal policymaking, raising questions about whose interests are most represented as hospital lobbying shapes health policy.

## Notes

Supplement 1. eTable 1. All hospital industry organizations with 2024 lobbying spend, categorized by type. eTable 2. Lobbying expenditures for national hospital associations, 2024 eTable 3. Lobbying expenditures for state and regional hospital associations, 2024 eTable 4. Hospital association lobbying expenditures per hospital, by state, 2024 eFigure. Intensity of federal lobbying by state hospital associations, 2024 eReferences

Supplement 2. Data Sharing Statement
