# Supplementary material for: Patterns of Federal Lobbying by the Hospital Industry
Source: JAMA Health Forum. 2026 Mar 13;7(3):e260117. doi: 10.1001/jamahealthforum.2026.0117 (PMC12988440; doi:10.1001/jamahealthforum.2026.0117)
Supplement: Supplement 1. — eTable 1. All hospital industry organizations with 2024 lobbying spend, categorized by type. eTable 2. Lobbying expenditures for national hospital associations, 2024 eTable 3. Lobbying expenditures for state and regional hospital associations, 2024 eTable 4. Hospital association lobbying expenditures per hospital, by state, 2024 eFigure. Intensity of federal lobbying by state hospital associations, 2024 eReferences [file jamahealthforum-e260117-s001.pdf]

# Supplemental Online Content

Korostoff-Larsson O, Shore C, Taylor LA. Patterns of federal lobbying by the hospital industry. *JAMA Health Forum*. 2026;7(3):e260117. doi:10.1001/jamahealthforum.2026.0117

**eTable 1.** All hospital industry organizations with 2024 lobbying spend, categorized by type
**eTable 2.** Lobbying expenditures for national hospital associations, 2024
**eTable 3.** Lobbying expenditures for state and regional hospital associations, 2024
**eTable 4.** Hospital association lobbying expenditures per hospital, by state, 2024
**eFigure.** Intensity of federal lobbying by state hospital associations, 2024
**eReferences**

This supplemental material has been provided by the authors to give readers additional information about their work.

© 2026 Korostoff-Larsson O et al. *JAMA Health Forum*.

**eTable 1. All hospital industry organizations with 2024 lobbying spend, categorized by type**

| Organization | Subsidiaries | Type | Federal Lobbying Spend 2024 |
|---|---|---|---|
| American Hospital Assn | | National Hospital Association | $24,110,000.00 |
| Select Medical Holdings | Select Medical Corp | For-Profit Hospital System | $5,500,000.00 |
| Children's Hospital Assn | | National Hospital Association | $4,170,000.00 |
| HCA Inc | HCA Management Services | For-Profit Hospital System | $3,040,000.00 |
| Advocate Health | Atrium Health, Atrium Health Wake Forest Baptist | Non-Profit Hospital System | $2,420,000.00 |
| Federation of American Hospitals | | National Hospital Association | $2,380,000.00 |
| Tenet Healthcare | | For-Profit Hospital System | $2,200,000.00 |
| Ascension Health | | Non-Profit Hospital System | $2,163,000.00 |
| America's Essential Hospitals | | National Hospital Association | $1,960,000.00 |
| Greater New York Hospital Assn | | State Level Hospital Association | $1,890,000.00 |
| Trinity Health | Baycare Health System | Non-Profit Hospital System | $1,646,232.00 |
| Ochsner Health System | Ochsner Clinic Foundation | Non-Profit Hospital System | $1,630,000.00 |
| California Hospital Assn | | State Level Hospital Association | $1,530,000.00 |
| Mass General Brigham | | Non-Profit Hospital System | $1,223,000.00 |
| Encompass Health | | For-Profit Hospital System | $1,195,000.00 |
| Apollo Global Management | Lifepoint Health, Kindred Healthcare | PE-Owned Hospital System | $1,156,000.00 |
| Mayo Clinic | | Non-Profit Hospital System | $1,050,000.00 |
| UPMC Health System | University of Pittsburgh Medical Center | Non-Profit Hospital System | $1,030,000.00 |
| Northwell Health | Maimonides Medical Center | Non-Profit Hospital System | $970,000.00 |
| Nemours Foundation | | Non-Profit Hospital System | $962,159.00 |
| Sanford Health | Evangelical Lutheran Good Samaritan Soc | Non-Profit Hospital System | $960,000.00 |
| Corewell Health | Lakeland Regional Health | Non-Profit Hospital System | $750,000.00 |
| Hackensack Meridian Health | | Non-Profit Hospital System | $720,000.00 |
| New York-Presbyterian Hospital | | Non-Profit Hospital System | $690,000.00 |
| Catholic Health Assn of the US | | National Hospital Association | $639,307.00 |

© 2026 Korostoff-Larsson O et al. *JAMA Health Forum*.

| | | | |
|---|---|---|---|
| Children's Hospital of Philadelphia | | Non-Profit Hospital System | $580,000.00 |
| Loma Linda University Health | | Non-Profit Hospital System | $560,000.00 |
| Memorial Sloan-Kettering Cancer Center | | Non-Profit Hospital System | $560,000.00 |
| MetroHealth System | | Non-Profit Hospital System | $530,000.00 |
| Sutter Health | | Non-Profit Hospital System | $530,000.00 |
| Cleveland Clinic | | Non-Profit Hospital System | $500,000.00 |
| Indiana University Health | | Non-Profit Hospital System | $500,000.00 |
| Providence St Joseph Health | | Non-Profit Hospital System | $480,000.00 |
| Alliance of Dedicated Cancer Centers | | National Hospital Association | $478,000.00 |
| Community Health Systems | | For-Profit Hospital System | $470,000.00 |
| University of Texas/Southwestern Medical Center | | Non-Profit Hospital System | $468,148.00 |
| Boston Children's Hospital | | Non-Profit Hospital System | $460,000.00 |
| Christus Health | | Non-Profit Hospital System | $440,000.00 |
| Fred Hutchinson Cancer Center | | Non-Profit Hospital System | $440,000.00 |
| Montefiore Health System | | Non-Profit Hospital System | $440,000.00 |
| Ballad Health | | Non-Profit Hospital System | $420,000.00 |
| CommonSpirit Health | CHI Memorial, Chi St Vincent | Non-Profit Hospital System | $420,000.00 |
| Healthcare Assn of New York State | | State Level Hospital Association | $410,000.00 |
| Alliance for Rural Hospital Access | | National Hospital Association | $380,000.00 |
| Mount Sinai Medical Center | | Non-Profit Hospital System | $380,000.00 |
| Cedars-Sinai Medical Center | | Non-Profit Hospital System | $370,000.00 |
| Children's Healthcare of Atlanta | | Non-Profit Hospital System | $370,000.00 |
| Children's Health System of Texas | | Non-Profit Hospital System | $360,000.00 |
| Front Line Hospital Alliance | | National Hospital Association | $360,000.00 |
| Seattle Children's | | Non-Profit Hospital System | $360,000.00 |
| Texas Children's Hospital | | Non-Profit Hospital System | $360,000.00 |
| Illinois Health & Hospital Assn | | State Level Hospital Association | $350,000.00 |
| Children's Hospital & Health System | | Non-Profit Hospital System | $340,000.00 |
| Children's Hospital Colorado | | Non-Profit Hospital System | $340,000.00 |
| Florida Hospital Assn | | State Level Hospital Association | $331,481.00 |

© 2026 Korostoff-Larsson O et al. *JAMA Health Forum*.

| | | | |
|---|---|---|---|
| Equity Group Investments | Ardent Health Services | PE-Owned Hospital System | $320,000.00 |
| Roper St Francis Healthcare | | Non-Profit Hospital System | $320,000.00 |
| University of Minnesota Health | Fairview Health Services | Non-Profit Hospital System | $320,000.00 |
| Doctors Hospital at Renaissance | | For-Profit Hospital System | $310,000.00 |
| Missouri Hospital Assn | | State Level Hospital Association | $310,000.00 |
| Rochester Regional Health | | Non-Profit Hospital System | $310,000.00 |
| Iroquois Healthcare Alliance | | State Level Hospital Association | $300,000.00 |
| UT MD Anderson Cancer Center | | Non-Profit Hospital System | $300,000.00 |
| Jefferson Health System | Main Line Health, Thomas Jefferson University Hospital, Magee Rehabilitation Hospital, Lehigh Valley Health Network | Non-Profit Hospital System | $284,525.00 |
| OU Medicine | | Non-Profit Hospital System | $280,000.00 |
| University of Wisconsin Medical Foundation | | Non-Profit Hospital System | $280,000.00 |
| Virginia Hospital & Healthcare Assn | | State Level Hospital Association | $280,000.00 |
| Dana-Farber Cancer Institute | | Non-Profit Hospital System | $271,942.00 |
| AnMed Health | | Non-Profit Hospital System | $270,000.00 |
| Hospital & Healthsystem Assn of Pennsylvania | | State Level Hospital Association | $270,000.00 |
| Texas Essential Healthcare Partnerships | | State Level Hospital Association | $270,000.00 |
| Geisinger Health Systems | Geisinger Health System Foundation | Non-Profit Hospital System | $262,339.00 |
| Moffitt Cancer Center | | Non-Profit Hospital System | $260,000.00 |
| Steward Health Care | | For-Profit Hospital System | $260,000.00 |
| University of Chicago Medical Center | | Non-Profit Hospital System | $255,000.00 |
| AdventHealth | | Non-Profit Hospital System | $246,126.00 |
| Boston Medical Center | | Non-Profit Hospital System | $240,000.00 |
| Florida Essential Healthcare Partnerships | | State Level Hospital Association | $240,000.00 |
| Lurie Children's Hospital of Chicago | | Non-Profit Hospital System | $240,000.00 |
| Medstar Health | | Non-Profit Hospital System | $240,000.00 |
| National Assn of Freestanding Emergency Centers | | National Hospital Association | $240,000.00 |

© 2026 Korostoff-Larsson O et al. *JAMA Health Forum*.

| | | | |
|---|---|---|---|
| OSF Healthcare System | | Non-Profit Hospital System | $240,000.00 |
| Shirley Ryan AbilityLab | | Non-Profit Hospital System | $240,000.00 |
| Spartanburg Regional Healthcare System | | Non-Profit Hospital System | $240,000.00 |
| Thomas Health System | | Non-Profit Hospital System | $240,000.00 |
| Tufts Medicine | | Non-Profit Hospital System | $240,000.00 |
| UAB Health System | | Non-Profit Hospital System | $240,000.00 |
| UnityPoint Health | | Non-Profit Hospital System | $240,000.00 |
| Texas Health Resources | | Non-Profit Hospital System | $238,400.00 |
| Nationwide Children's Hospital | | Non-Profit Hospital System | $235,000.00 |
| Adventist Healthcare | | Non-Profit Hospital System | $230,000.00 |
| Intermountain Health Care | | Non-Profit Hospital System | $230,000.00 |
| Vanderbilt University Medical Center | | Non-Profit Hospital System | $230,000.00 |
| Iowa Hospital Assn | | State Level Hospital Association | $228,515.00 |
| Post Acute Medical | | For-Profit Hospital System | $225,490.00 |
| KPC Global Hospitals | | For-Profit Hospital System | $225,000.00 |
| Essentia Health | | Non-Profit Hospital System | $220,000.00 |
| Fresno Community Hospital & Medical Center | | Non-Profit Hospital System | $210,000.00 |
| Southcentral Foundation | | Non-Profit Hospital System | $210,000.00 |
| Nuvance Health | | Non-Profit Hospital System | $209,400.00 |
| CentraCare Health | | Non-Profit Hospital System | $208,000.00 |
| Adventist Health Policy Assn | | National Hospital Association | $200,000.00 |
| BJC Healthcare | | Non-Profit Hospital System | $200,000.00 |
| Children's National Health System | | Non-Profit Hospital System | $200,000.00 |
| City of Hope | | Non-Profit Hospital System | $200,000.00 |
| Gundersen Health System | Gundersen Lutheran Administrative Services | Non-Profit Hospital System | $200,000.00 |
| Health & Hospital Corp of Marion County | | Non-Profit Hospital System | $200,000.00 |
| Houston Methodist | | Non-Profit Hospital System | $200,000.00 |
| Johns Hopkins Health System | Kennedy Krieger Institute | Non-Profit Hospital System | $200,000.00 |
| Knoxville Area Hospital Alliance | | State Level Hospital Association | $200,000.00 |
| Lifebridge Health | | Non-Profit Hospital System | $200,000.00 |
| Michigan Health & Hospital Assn | | State Level Hospital Association | $200,000.00 |
| Phoebe Putney Health System | | Non-Profit Hospital System | $200,000.00 |
| Physician-Led Healthcare for America | | National Hospital Association | $200,000.00 |

© 2026 Korostoff-Larsson O et al. *JAMA Health Forum*.

| | | | |
|---|---|---|---|
| Professional Hospital Guaynabo | | Non-Profit Hospital System | $200,000.00 |
| Regional One Health | | Non-Profit Hospital System | $200,000.00 |
| Safety Net Hospital Alliance of Florida | | State Level Hospital Association | $200,000.00 |
| Sun River Health | | Non-Profit Hospital System | $200,000.00 |
| Tam General Hospital | | Non-Profit Hospital System | $200,000.00 |
| Texas Hospital Assn | | State Level Hospital Association | $200,000.00 |
| Trauma Center Assn of America | | National Hospital Association | $200,000.00 |
| UC Health | | Non-Profit Hospital System | $200,000.00 |
| WVU Medicine | Garrett Regional Medical Center, Potomac Valley Hospital | Non-Profit Hospital System | $200,000.00 |
| ProMedica Health System | | Non-Profit Hospital System | $196,000.00 |
| Allina Health | | Non-Profit Hospital System | $190,000.00 |
| California Children's Hospital Assn | | State Level Hospital Association | $180,000.00 |
| Hackensack University Health Network | Hackensack University Medical Center | Non-Profit Hospital System | $180,000.00 |
| Henry Ford Health System | | Non-Profit Hospital System | $180,000.00 |
| Southcoast Health System | | Non-Profit Hospital System | $180,000.00 |
| Valley Presbyterian Hospital | | Non-Profit Hospital System | $180,000.00 |
| VHC Health | | Non-Profit Hospital System | $180,000.00 |
| Wellspan Health | | Non-Profit Hospital System | $170,000.00 |
| Community Health Network (Indiana) | | Non-Profit Hospital System | $168,000.00 |
| Allegiance Health Management | | For-Profit Hospital System | $160,000.00 |
| Children's Hospital Los Angeles | | Non-Profit Hospital System | $160,000.00 |
| Children's Hospital of the King's Daughters | | Non-Profit Hospital System | $160,000.00 |
| Denver Health & Hospital Authority | | Non-Profit Hospital System | $160,000.00 |
| Erlanger Health System | | Non-Profit Hospital System | $160,000.00 |
| Kentucky Hospital Assn | | State Level Hospital Association | $160,000.00 |
| MediSys Health Network | | Non-Profit Hospital System | $160,000.00 |
| Memorial Health System (Illinois) | | Non-Profit Hospital System | $160,000.00 |
| National Jewish Health | | Non-Profit Hospital System | $160,000.00 |
| New Jersey Hospital Assn | | State Level Hospital Association | $160,000.00 |
| Nicklaus Children's Health System | | Non-Profit Hospital System | $160,000.00 |
| RWJBarnabas Health | | Non-Profit Hospital System | $160,000.00 |

© 2026 Korostoff-Larsson O et al. *JAMA Health Forum*.

| | | | |
|---|---|---|---|
| Temple University Health System | | Non-Profit Hospital System | $160,000.00 |
| University of Tennessee Medical Center | | Non-Profit Hospital System | $160,000.00 |
| Valley Health System | Valley Hospital | Non-Profit Hospital System | $160,000.00 |
| Private Essential Access Community Hosp | | State Level Hospital Association | $159,556.00 |
| National Alliance of Safety-Net Hospitals | | National Hospital Association | $156,000.00 |
| Suburban Health Organization | | State Level Hospital Association | $156,000.00 |
| Ellis Hospital | | Non-Profit Hospital System | $150,000.00 |
| Piedmont Healthcare | | Non-Profit Hospital System | $150,000.00 |
| Tidelands Health | | Non-Profit Hospital System | $150,000.00 |
| Wisconsin Hospital Assn | | State Level Hospital Association | $150,000.00 |
| Baptist Healthcare System | | Non-Profit Hospital System | $148,000.00 |
| Baylor Scott & White Health | | Non-Profit Hospital System | $144,000.00 |
| Medical University Hospital Authority | | Non-Profit Hospital System | $140,000.00 |
| National Assn of Long Term Hospitals | | National Hospital Association | $140,000.00 |
| Queen's Health Systems | | Non-Profit Hospital System | $139,500.00 |
| Cincinnati Children's Hospital Medical Center | | Non-Profit Hospital System | $138,684.00 |
| Franciscan Missionaries of Our Lady Health System | | Non-Profit Hospital System | $130,000.00 |
| Indiana Hospital Assn | | State Level Hospital Association | $130,000.00 |
| North Carolina Healthcare Assn | | State Level Hospital Association | $130,000.00 |
| SSM Health | | Non-Profit Hospital System | $130,000.00 |
| Wellstar Health System | | Non-Profit Hospital System | $130,000.00 |
| Parkview Health System | | Non-Profit Hospital System | $126,000.00 |
| Coalition of Long Term Acute Care Hospitals | | National Hospital Association | $125,770.00 |
| Alaska Native Tribal Health Consortium | | Non-Profit Hospital System | $125,000.00 |
| Summa Health System | | Non-Profit Hospital System | $124,000.00 |
| Kaiser Permanente | Maui Health System | Non-Profit Hospital System | $123,200.00 |
| Advanced Diagnostics Healthcare System | | For-Profit Hospital System | $120,000.00 |
| Avera Health | | Non-Profit Hospital System | $120,000.00 |
| Baystate Health Systems | | Non-Profit Hospital System | $120,000.00 |
| Billings Clinic | | Non-Profit Hospital System | $120,000.00 |
| Cape Cod Healthcare | | Non-Profit Hospital System | $120,000.00 |

© 2026 Korostoff-Larsson O et al. *JAMA Health Forum*.

| | | | |
|---|---|---|---|
| Catholic Health Services of Long Island | | Non-Profit Hospital System | $120,000.00 |
| Children's Hospital Assn of Texas | | State Level Hospital Association | $120,000.00 |
| Community Hospital (Colorado) | | Non-Profit Hospital System | $120,000.00 |
| Ephraim McDowell Health | | Non-Profit Hospital System | $120,000.00 |
| Fairfield Medical Center | | Non-Profit Hospital System | $120,000.00 |
| Grande Ronde Hospital | | Non-Profit Hospital System | $120,000.00 |
| Hocking Valley Community Hospital | | Non-Profit Hospital System | $120,000.00 |
| LaSalle General Hospital | | Non-Profit Hospital System | $120,000.00 |
| MercyOne | MercyOne Des Moines Medical Center | Non-Profit Hospital System | $120,000.00 |
| Midwest City Memorial Hospital Authority | | Non-Profit Hospital System | $120,000.00 |
| Mount Sinai Health System | Brooklyn Hospital Center | Non-Profit Hospital System | $120,000.00 |
| Multicare Health System | | Non-Profit Hospital System | $120,000.00 |
| Neosho Memorial Regional Medical Center | | Non-Profit Hospital System | $120,000.00 |
| Northwestern Memorial Healthcare | | Non-Profit Hospital System | $120,000.00 |
| Pam Health | | For-Profit Hospital System | $120,000.00 |
| Rady Children's Hospital San Diego | | Non-Profit Hospital System | $120,000.00 |
| RiverBridge Specialty Hospital | | For-Profit Hospital System | $120,000.00 |
| SouthEast Alaska Regional Health Consortium | | Non-Profit Hospital System | $120,000.00 |
| St Charles Health System | | Non-Profit Hospital System | $120,000.00 |
| St Joseph's Regional Medical Center | | Non-Profit Hospital System | $120,000.00 |
| Swedish American Health System | | Non-Profit Hospital System | $120,000.00 |
| Tennessee Hospital Assn | | State Level Hospital Association | $120,000.00 |
| UMass Memorial Health Care | | Non-Profit Hospital System | $120,000.00 |
| Universal Health Services | | For-Profit Hospital System | $120,000.00 |
| University Hospitals | | Non-Profit Hospital System | $120,000.00 |
| University of Kansas Hospital Authority | | Non-Profit Hospital System | $120,000.00 |
| UofL Health | | Non-Profit Hospital System | $120,000.00 |
| Valley Children's Healthcare | | Non-Profit Hospital System | $120,000.00 |

© 2026 Korostoff-Larsson O et al. *JAMA Health Forum*.

| | | | |
|---|---|---|---|
| Van Buren County Hospital | | Non-Profit Hospital System | $120,000.00 |
| Mercy Health System | | Non-Profit Hospital System | $117,500.00 |
| Atlantic Health System | | Non-Profit Hospital System | $110,000.00 |
| Baptist Health (Florida) | | Non-Profit Hospital System | $110,000.00 |
| Bon Secours Mercy Health | | Non-Profit Hospital System | $110,000.00 |
| Hale Makua Health Services | | Non-Profit Hospital System | $110,000.00 |
| Prospect Medical Holdings | CharterCARE Health Partners | PE-Owned Hospital System | $110,000.00 |
| District Hospital Leadership Forum | | State Level Hospital Association | $108,596.00 |
| Capital Health System | | Non-Profit Hospital System | $100,000.00 |
| NYU Langone Medical Center | | Non-Profit Hospital System | $100,000.00 |
| Premier Health Group (California) | | For-Profit Hospital System | $100,000.00 |
| Ridgecrest Regional Hospital | | Non-Profit Hospital System | $100,000.00 |
| Rush System for Health | | Non-Profit Hospital System | $100,000.00 |
| St Jude Children's Research Hospital | | Non-Profit Hospital System | $100,000.00 |
| Stilwell Memorial Hospital | | Non-Profit Hospital System | $100,000.00 |
| Asociacion de Hospitales de Puerto Rico | | State Level Hospital Association | $96,000.00 |
| Aultman Health Foundation | Aultman Hospital | Non-Profit Hospital System | $96,000.00 |
| Legacy Health System | | Non-Profit Hospital System | $96,000.00 |
| Yuma Regional Medical Center | | Non-Profit Hospital System | $92,700.00 |
| Indiana Regional Medical Center | | Non-Profit Hospital System | $90,000.00 |
| Memorial Health System (Mississippi) | Memorial Hospital at Gulfport | Non-Profit Hospital System | $90,000.00 |
| Methodist Le Bonheur Healthcare | | Non-Profit Hospital System | $90,000.00 |
| Holy Name Medical Center | | Non-Profit Hospital System | $85,000.00 |
| Lee Health | | Non-Profit Hospital System | $85,000.00 |
| University of Pennsylvania Health System | | Non-Profit Hospital System | $83,400.00 |
| Acadiana Management Group | | For-Profit Hospital System | $80,000.00 |
| Adirondack Health | | Non-Profit Hospital System | $80,000.00 |
| Alabama Hospital Assn | | State Level Hospital Association | $80,000.00 |
| AtlantiCare Health System | | Non-Profit Hospital System | $80,000.00 |
| Banner Health | | Non-Profit Hospital System | $80,000.00 |
| Beth Israel Lahey Health | | Non-Profit Hospital System | $80,000.00 |

© 2026 Korostoff-Larsson O et al. *JAMA Health Forum*.

| | | | |
|---|---|---|---|
| Boone Memorial Health | | Non-Profit Hospital System | $80,000.00 |
| Citizens Medical Center (Louisiana) | | Non-Profit Hospital System | $80,000.00 |
| Colorado Hospital Assn | | State Level Hospital Association | $80,000.00 |
| Community Foundation of Northwest Indiana | | Non-Profit Hospital System | $80,000.00 |
| Covenant Health System | | Non-Profit Hospital System | $80,000.00 |
| Cox Health Systems | | Non-Profit Hospital System | $80,000.00 |
| Crawford Memorial Hospital | | Non-Profit Hospital System | $80,000.00 |
| Crisp Regional Health Services | | Non-Profit Hospital System | $80,000.00 |
| Deborah Heart & Lung Center | | Non-Profit Hospital System | $80,000.00 |
| Franciscan Alliance | | Non-Profit Hospital System | $80,000.00 |
| Garnet Health | | Non-Profit Hospital System | $80,000.00 |
| Gateways Hospital & Mental Health Center | | Non-Profit Hospital System | $80,000.00 |
| Golden Valley Memorial Healthcare | | Non-Profit Hospital System | $80,000.00 |
| Holzer Health System | | Non-Profit Hospital System | $80,000.00 |
| Hospital Assn of Oregon | | State Level Hospital Association | $80,000.00 |
| Hospital for Special Surgery | | Non-Profit Hospital System | $80,000.00 |
| Huntsville Hospital | | Non-Profit Hospital System | $80,000.00 |
| Jackson Parish Hospital | | Non-Profit Hospital System | $80,000.00 |
| JPS Health Network | | Non-Profit Hospital System | $80,000.00 |
| Kona Community Hospital | | Non-Profit Hospital System | $80,000.00 |
| Larkin Health System | | For-Profit Hospital System | $80,000.00 |
| Mary Bird Perkins Cancer Center | | Non-Profit Hospital System | $80,000.00 |
| Massachusetts Health & Hospital Assn | | State Level Hospital Association | $80,000.00 |
| McLaren Health Care | | Non-Profit Hospital System | $80,000.00 |
| MLK Community Healthcare | | Non-Profit Hospital System | $80,000.00 |
| Mosaic Life Care | | Non-Profit Hospital System | $80,000.00 |
| Mountain Health Network | | Non-Profit Hospital System | $80,000.00 |
| Natchitoches Regional Medical Center | | Non-Profit Hospital System | $80,000.00 |
| North Arkansas Regional Medical Center | | Non-Profit Hospital System | $80,000.00 |
| Oneida Healthcare Center | | Non-Profit Hospital System | $80,000.00 |
| Parker Jewish Institute | | Non-Profit Hospital System | $80,000.00 |
| Pikeville Medical Center | | Non-Profit Hospital System | $80,000.00 |
| Priority Hospital Group | | Non-Profit Hospital System | $80,000.00 |

| | | | |
|---|---|---|---|
| Rhode Island Hospital Assn | | State Level Hospital Association | $80,000.00 |
| Spanish Peaks Regional Health Center | | Non-Profit Hospital System | $80,000.00 |
| St Anthony Hospital | | Non-Profit Hospital System | $80,000.00 |
| Stillwater Medical Center | | Non-Profit Hospital System | $80,000.00 |
| Virtua Health | | Non-Profit Hospital System | $80,000.00 |
| Washington State Hospital Assn | | State Level Hospital Association | $80,000.00 |
| Wayne County Hospital (Iowa) | | Non-Profit Hospital System | $80,000.00 |
| Westchester Medical Center | | Non-Profit Hospital System | $80,000.00 |
| Wooster Community Hospital | | Non-Profit Hospital System | $80,000.00 |
| Wyandot Memorial Hospital | | Non-Profit Hospital System | $80,000.00 |
| Zuckerberg San Francisco General Hospital | San Francisco General Hospital Foundation | Non-Profit Hospital System | $80,000.00 |
| Kansas Hospital Assn | | State Level Hospital Association | $76,000.00 |
| East Adams Rural Healthcare | | Non-Profit Hospital System | $75,000.00 |
| Blessing Health System | | Non-Profit Hospital System | $72,000.00 |
| Craig Hospital | | Non-Profit Hospital System | $72,000.00 |
| Shepherd Center | | Non-Profit Hospital System | $72,000.00 |
| Southeast Georgia Health System | | Non-Profit Hospital System | $72,000.00 |
| Appalachian Regional Healthcare | | Non-Profit Hospital System | $70,000.00 |
| Tuba City Regional Health Care Corp | | Non-Profit Hospital System | $66,000.00 |
| Eisenhower Health | | Non-Profit Hospital System | $65,000.00 |
| Moses/Weitzman Health System | | Non-Profit Hospital System | $65,000.00 |
| Association of Cancer Care Centers | | National Hospital Association | $60,000.00 |
| Coulee Medical Center | | Non-Profit Hospital System | $60,000.00 |
| Cullman Regional Medical Center | | Non-Profit Hospital System | $60,000.00 |
| Guam Regional Medical City | | Non-Profit Hospital System | $60,000.00 |
| Jackson Health System | | Non-Profit Hospital System | $60,000.00 |
| Ohio Children's Hospital Assn | | State Level Hospital Association | $60,000.00 |
| Oklahoma Heart Hospital | | Non-Profit Hospital System | $60,000.00 |
| Singing River Hospital System | | Non-Profit Hospital System | $60,000.00 |
| Stanisaluas Surgical Hospital | | For-Profit Hospital System | $60,000.00 |
| Calvary Hospital | | Non-Profit Hospital System | $50,000.00 |
| Guthrie Clinic | | Non-Profit Hospital System | $48,069.00 |

© 2026 Korostoff-Larsson O et al. *JAMA Health Forum*.

| | | | |
|---|---|---|---|
| Centura Health | | Non-Profit Hospital System | $48,000.00 |
| Wray Community Hospital | | Non-Profit Hospital System | $48,000.00 |
| Benefis Health System | | Non-Profit Hospital System | $45,000.00 |
| Great Falls Clinic & Hospital | | Non-Profit Hospital System | $45,000.00 |
| Peace Health | | Non-Profit Hospital System | $45,000.00 |
| Whitfield Hospital | | Non-Profit Hospital System | $45,000.00 |
| Astria Health | | Non-Profit Hospital System | $40,000.00 |
| Community Memorial Hospital (Ohio) | | Non-Profit Hospital System | $40,000.00 |
| Dartmouth-Hitchcock Medical Center | | Non-Profit Hospital System | $40,000.00 |
| Dimock Center | | Non-Profit Hospital System | $40,000.00 |
| Elizabeth Seton Children's | | Non-Profit Hospital System | $40,000.00 |
| Eskenazi Health | | Non-Profit Hospital System | $40,000.00 |
| Garfield County Hospital District | | Non-Profit Hospital System | $40,000.00 |
| Harris Health System | | Non-Profit Hospital System | $40,000.00 |
| Hospital Alliance of New Jersey | | State Level Hospital Association | $40,000.00 |
| Inspira Health Network | | Non-Profit Hospital System | $40,000.00 |
| Island Health | | Non-Profit Hospital System | $40,000.00 |
| Jefferson Healthcare | | Non-Profit Hospital System | $40,000.00 |
| Nassau-Suffolk Hospital Council | | State Level Hospital Association | $40,000.00 |
| National Assn of Epilepsy Centers | | National Hospital Association | $40,000.00 |
| Ohio Hospital Assn | | State Level Hospital Association | $40,000.00 |
| Olympic Medical Center | | Non-Profit Hospital System | $40,000.00 |
| Petersburg Medical Center | | Non-Profit Hospital System | $40,000.00 |
| Public Health Trust/Jackson Health System | | Non-Profit Hospital System | $40,000.00 |
| Samaritan Healthcare | | Non-Profit Hospital System | $40,000.00 |
| St Luke's Health System (Idaho) | | Non-Profit Hospital System | $40,000.00 |
| St Luke's University Health Network | | Non-Profit Hospital System | $40,000.00 |
| Stanford Children's Health | | Non-Profit Hospital System | $40,000.00 |
| Stanford Health Care | | Non-Profit Hospital System | $40,000.00 |
| TidalHealth | | Non-Profit Hospital System | $40,000.00 |
| TriState Health | Tri-State Memorial Hospital | Non-Profit Hospital System | $40,000.00 |
| West Virginia Hospital Assn | | State Level Hospital Association | $40,000.00 |
| Texas Organization of Rural & Community Hospitals | | State Level Hospital Association | $32,400.00 |

© 2026 Korostoff-Larsson O et al. *JAMA Health Forum*.

| | | | |
|---|---|---|---|
| Arkansas Children's Hospital | | Non-Profit Hospital System | $30,000.00 |
| Franklin Medical Center | | Non-Profit Hospital System | $30,000.00 |
| Intensive Specialty Hospital | | For-Profit Hospital System | $30,000.00 |
| Mid Coast Health System | | Non-Profit Hospital System | $30,000.00 |
| Nebraska Hospital Assn | | State Level Hospital Association | $30,000.00 |
| Northern Arizona Healthcare | | Non-Profit Hospital System | $30,000.00 |
| LifeCare 2.0 LLC | | For-Profit Hospital System | $27,000.00 |
| Lincoln Community Hospital & Care Center | | Non-Profit Hospital System | $24,000.00 |
| Insight Institute of Neurosurgery & Neuroscience | | Non-Profit Hospital System | $22,000.00 |
| Cerberus Capital Management | St Elizabeth's Medical Center (MA) | PE-Owned Hospital System | $20,000.00 |
| Conway Medical Center | | Non-Profit Hospital System | $20,000.00 |
| Grady Health System | | Non-Profit Hospital System | $20,000.00 |
| Lifespan (Rhode Island) | | Non-Profit Hospital System | $20,000.00 |
| Northern Nye County Hospital District | | State Level Hospital Association | $20,000.00 |
| Orlando Health | | Non-Profit Hospital System | $20,000.00 |
| University Health System (Tennessee) | | Non-Profit Hospital System | $20,000.00 |
| Children's Mercy Hospital | | Non-Profit Hospital System | $15,000.00 |
| Bassett Healthcare Network | | Non-Profit Hospital System | $10,000.00 |
| Forrest General Hospital | | Non-Profit Hospital System | $10,000.00 |
| Rock Regional Hospital | | For-Profit Hospital System | $10,000.00 |
| Rush University Medical Center | | Non-Profit Hospital System | $7,500.00 |
| ChristianaCare | | Non-Profit Hospital System | $5,807.00 |

## Definitions of Organization Types

| Type of Organization | Description |
|---|---|
| Nonprofit Hospital or Health System | Hospitals and health systems operating under 501(c)(3) nonprofit tax status. Includes public hospitals. |
| For-Profit Hospital or Health System | Hospitals or health systems organized as for-profit corporations. |
| Private Equity–Owned Hospital or Health System | Hospitals or health systems owned in majority by private equity firms involved in healthcare acquisitions or financing. |

© 2026 Korostoff-Larsson O et al. *JAMA Health Forum*.

| | |
|---|---|
| National Hospital Association | Trade associations representing hospitals or health systems nationwide, often with a specific characteristic (e.g., pediatric hospitals, rural hospitals). |
| State or Regional Hospital Association | Associations representing hospitals within a single state or a regional cluster. |

© 2026 Korostoff-Larsson O et al. *JAMA Health Forum*.

## eTable 2. Lobbying expenditures for national hospital associations, 2024

| Association | Who They Represent | 2024 Lobbying Spend |
|---|---|---|
| American Hospital Assn | Broadly U.S. hospitals and health systems | $24,110,000.00 |
| Children's Hospital Assn | Pediatric hospitals | $4,170,000.00 |
| Federation of American Hospitals | For-profit hospitals | $2,380,000.00 |
| America's Essential Hospitals | Safety-net hospitals | $1,960,000.00 |
| Catholic Health Assn of the US | Catholic-affiliated health systems | $639,307.00 |
| Alliance of Dedicated Cancer Centers | Independent cancer centers | $478,000.00 |
| Alliance for Rural Hospital Access | Rural hospitals | $380,000.00 |
| Front Line Hospital Alliance | Urban “super” safety-net hospitals (≥40% Medicaid/uninsured) | $360,000.00 |
| National Assn of Freestanding Emergency Centers | Freestanding emergency departments | $240,000.00 |
| Adventist Health Policy Assn | Faith-based Adventist health systems | $200,000.00 |
| Physician-Led Healthcare for America | Physician-owned hospitals | $200,000.00 |
| Trauma Center Assn of America | Trauma centers | $200,000.00 |
| National Alliance of Safety-Net Hospitals | Public/nonprofit safety-net hospitals | $156,000.00 |
| National Assn of Long-Term Hospitals | Long-term acute care hospitals | $140,000.00 |
| Coalition of Long-Term Acute Care Hospitals | Long-term acute care hospitals | $125,770.00 |
| Association of Cancer Care Centers | Cancer treatment centers | $60,000.00 |
| National Assn of Epilepsy Centers | Epilepsy treatment centers | $40,000.00 |
| **Total** | - | $35,839,077.00 |

© 2026 Korostoff-Larsson O et al. *JAMA Health Forum*.

**eTable 3. Lobbying expenditures for state and regional hospital associations, 2024**

| Association | 2024 Lobbying Spend | Represented States |
|---|---|---|
| Greater New York Hospital Assn | $1,890,000.00 | NY, NJ, CT, RI |
| California Hospital Assn | $1,530,000.00 | CA |
| Healthcare Assn of New York State | $410,000.00 | NY |
| Illinois Health & Hospital Assn | $350,000.00 | IL |
| Florida Hospital Assn | $331,481.00 | FL |
| Missouri Hospital Assn | $310,000.00 | MO |
| Iroquois Healthcare Alliance (New York) | $300,000.00 | NY |
| Virginia Hospital & Healthcare Assn | $280,000.00 | VA |
| Hospital & Health System Assn of Pennsylvania | $270,000.00 | PA |
| Texas Essential Healthcare Partnerships | $270,000.00 | TX |
| Florida Essential Healthcare Partnerships | $240,000.00 | FL |
| Iowa Hospital Assn | $228,515.00 | IA |
| Knoxville Area Hospital Alliance (Tennessee) | $200,000.00 | TN |
| Michigan Health & Hospital Assn | $200,000.00 | MI |
| Safety Net Hospital Alliance of Florida | $200,000.00 | FL |
| Texas Hospital Assn | $200,000.00 | TX |
| California Children's Hospital Assn | $180,000.00 | CA |
| Kentucky Hospital Assn | $160,000.00 | KY |
| New Jersey Hospital Assn | $160,000.00 | NJ |
| Private Essential Access Community Hosp (California) | $159,556.00 | CA |
| Suburban Health Organization (Indiana) | $156,000.00 | IN |
| Wisconsin Hospital Assn | $150,000.00 | WI |
| Indiana Hospital Assn | $130,000.00 | IN |
| North Carolina Healthcare Assn | $130,000.00 | NC |
| Tennessee Hospital Assn | $120,000.00 | TN |
| Children's Hospital Assn of Texas | $120,000.00 | TX |
| District Hospital Leadership Forum (California) | $108,596.00 | CA |
| Asociacion de Hospitales de Puerto Rico | $96,000.00 | Puerto Rico |
| Alabama Hospital Assn | $80,000.00 | AL |
| Colorado Hospital Assn | $80,000.00 | CO |
| Hospital Assn of Oregon | $80,000.00 | OR |
| Massachusetts Health & Hospital Assn | $80,000.00 | MA |
| Rhode Island Hospital Assn | $80,000.00 | RI |
| Washington State Hospital Assn | $80,000.00 | WA |
| Kansas Hospital Assn | $76,000.00 | KS |
| Ohio Children's Hospital Assn | $60,000.00 | OH |
| Nassau-Suffolk Hospital Council (New York) | $40,000.00 | NY |
| Ohio Hospital Assn | $40,000.00 | OH |
| West Virginia Hospital Assn | $40,000.00 | WV |
| Hospital Alliance of New Jersey | $40,000.00 | NJ |
| Texas Organization of Rural & Community Hospitals | $32,400.00 | TX |
| Nebraska Hospital Assn | $30,000.00 | NE |
| Northern Nye County Hospital District (Nevada) | $20,000.00 | NV |
| **Total** | $9,738,548.00 | - |

© 2026 Korostoff-Larsson O et al. *JAMA Health Forum*.

## eTable 4. Hospital association lobbying expenditures per hospital, by state, 2024

| State[a] | Total Association Spending | No. of Hospitals[1] | Per Hospital Spend |
|---|---|---|---|
| Rhode Island | $1,970,000.00 | 11 | $179,090.91 |
| Connecticut | $1,890,000.00 | 34 | $55,588.24 |
| New Jersey | $2,090,000.00 | 76 | $27,500.00 |
| New York | $2,640,000.00 | 180 | $14,666.67 |
| Iowa | $228,515.00 | 37 | $6,176.08 |
| California | $1,978,152.00 | 331 | $5,976.29 |
| Missouri | $310,000.00 | 80 | $3,875.00 |
| Tennessee | $320,000.00 | 95 | $3,368.42 |
| Indiana | $286,000.00 | 101 | $2,831.68 |
| Virginia | $320,000.00 | 87 | $3,678.16 |
| Kentucky | $160,000.00 | 74 | $2,162.16 |
| Florida | $771,481.00 | 222 | $3,475.14 |
| Puerto Rico | $96,000.00 | 55 | $1,745.45 |
| Illinois | $350,000.00 | 136 | $2,573.53 |
| Kansas | $76,000.00 | 56 | $1,357.14 |
| Wisconsin | $150,000.00 | 84 | $1,785.71 |
| Pennsylvania | $270,000.00 | 177 | $1,525.42 |
| Texas | $622,400.00 | 372 | $1,673.12 |
| Michigan | $200,000.00 | 101 | $1,980.20 |
| Oregon | $80,000.00 | 35 | $2,285.71 |
| Alabama | $80,000.00 | 85 | $941.18 |
| Nebraska | $30,000.00 | 28 | $1,071.43 |
| Colorado | $80,000.00 | 60 | $1,333.33 |
| North Carolina | $130,000.00 | 110 | $1,181.82 |
| Massachusetts | $80,000.00 | 69 | $1,159.42 |
| Washington | $80,000.00 | 60 | $1,333.33 |
| Ohio | $100,000.00 | 142 | $704.23 |
| Nevada | $20,000.00 | 32 | $625.00 |

a. States not included in table had no reported state or regional association spending.

© 2026 Korostoff-Larsson O et al. *JAMA Health Forum*.

# eFigure. Intensity of federal lobbying by state hospital associations, 2024

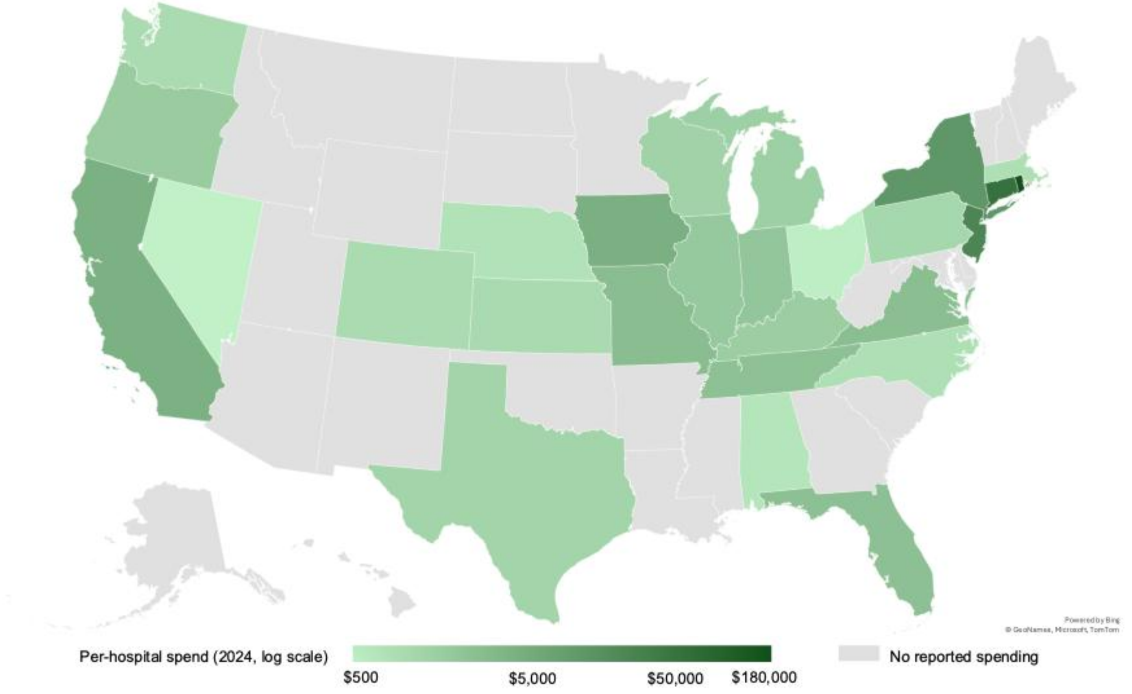


1. Data were visualized using Microsoft Excel (Version 16.89.1).
2. Figure includes spending by both state and regional associations.
3. Intensity of federal lobbying is measured on a per-hospital basis because some states have many more hospitals than others and therefore may spend more on hospital-related lobbying.
4. Spending is displayed on a $\log_{10}$ scale to account for the skewed distribution of spending and better reflect gradations in spending across states. Values ranged from $625 per hospital in Nevada to $179,091 per hospital in Rhode Island.

© 2026 Korostoff-Larsson O et al. *JAMA Health Forum*.

eReferences

1. American Hospital Directory. Hospital statistics by state. Accessed October 8, 2025. https://www.ahd.com/state_statistics.html

© 2026 Korostoff-Larsson O et al. *JAMA Health Forum*.
