# Supplementary material for: Patterns of Federal Lobbying by the Hospital Industry
Source: JAMA Health Forum. 2026 Mar 13;7(3):e260117. doi: 10.1001/jamahealthforum.2026.0117 (PMC12988440; doi:10.1001/jamahealthforum.2026.0117)
Supplement: Supplement 2. — Data Sharing Statement [file jamahealthforum-e260117-s002.pdf]

Data Sharing Statement

Korostoff-Larsson. Patterns of Federal Lobbying by the Hospital Industry. *JAMA Health Forum*. Published March 13, 2026. doi:10.1001/jamahealthforum.2026.0117

Data
Data available: Yes
Data types: Data (not involving human participants)
How to access data: Data available upon request to the corresponding author.
When available: With publication

Supporting Documents
Document types: None

Additional Information
Who can access the data: Anyone requesting the data
Types of analyses: Any purpose
Mechanisms of data availability: With approval of a proposal
